# Factors Associated with Nurses’ Intention to Leave Their Jobs after the Fukushima Daiichi Nuclear Power Plant Accident

**DOI:** 10.1371/journal.pone.0122389

**Published:** 2015-03-27

**Authors:** Yoshinobu Sato, Naomi Hayashida, Makiko Orita, Hideko Urata, Tetsuko Shinkawa, Yoshiko Fukushima, Yumiko Nakashima, Takashi Kudo, Shunichi Yamashita, Noboru Takamura

**Affiliations:** 1 Department of Global Health, Medicine, and Welfare, Atomic Bomb Disease Institute, Nagasaki University Graduate School of Biomedical Sciences, Nagasaki, Japan; 2 Fukushima Medical University Hospital, Fukushima, Japan; 3 Department of Nursing, Nagasaki University Graduate School of Biomedical Sciences, Nagasaki, Japan; 4 Hirosaki University School of Health Sciences, Hirosaki, Japan; 5 Department of Radioisotope Medicine, Atomic Bomb Disease Institute, Nagasaki University Graduate School of Biomedical Sciences, Nagasaki, Japan; 6 Department of Radiation Medical Sciences, Atomic Bomb Disease Institute, Nagasaki University Graduate School of Biomedical Sciences, Nagasaki, Japan; University of Pittsburgh, UNITED STATES

## Abstract

We conducted a survey among nurses who were working at the Fukushima Medical University Hospital at the time of the Fukushima Daiichi Nuclear Power Plant accident to clarify the factors associated with their intention to leave their jobs during the radiation emergency. We asked 345 nurses (17 men and 328 women) about their intention to leave their jobs after the accident. We also asked about relevant factors including the participants’ demographic factors, living situation, working status, and knowledge of radiation health effects. We found that living with preschoolers (OR = 1.87, 95%CI: 1.02–3.44, p = 0.042), anxiety about life in Fukushima City after the accident (OR = 5.55, 95%CI: 1.18–26.13, p = 0.030), consideration of evacuation from Fukushima after the accident (OR = 2.42, 95%CI: 1.45–4.06, p = 0.001), consideration of the possible radiation health effects in children (OR = 1.90, 95%CI: 1.02–3.44, p = 0.042), and anxiety about relationships with colleagues in the hospital after the accident (OR = 3.23, p = 0.001) were independently associated with the nurses’ intention to leave their jobs after the accident. On the other hand, the percentage of nurses with knowledge on radiation health effects was relatively low among those who had the intention to leave the job and among those who did not have the intention to leave the job after the accident, with no significant differences between the two groups. Our results suggest the need for an education program for nurses regarding radiation health effects.

## Introduction

The nuclear accident at the Fukushima Daiichi Nuclear Power Plant (FNPP) following the Great East Japan Earthquake occurred at 2:46 p.m. on March 11, 2011. At 8:50 p.m. of the same day, the governor of Fukushima Prefecture issued instructions for the evacuation of settlements within 2 km of the FNPP. At 9:23 p.m., the prime minister, in his capacity as director-general of the Nuclear Emergency Response Headquarters, ordered the evacuation of individuals within 3 km of the FNPP. He also ordered all individuals within 10 km to remain sheltered indoors. At 6:25 p.m. of the same day, the evacuation radius was expanded to 20 km. On March 15, 2011, instructions were issued for all people living between 20 km and 30 km from the FNPP to find shelter indoors [1–3]. As a result, almost 110,000 evacuated their hometowns. Many residents voluntarily evacuated outside of Fukushima Prefecture due to fear of radiation exposure. As of August 2014, 47,149 residents of Fukushima Prefecture remain evacuees in other prefectures [4].

After the hydrogen explosion at Units 1, 3, and 4 and the opening of the vents at Units 1, 2, and 3 of the FNPP, the ambient dose rate in Fukushima City reached 24.18 μSv/hr on March 15, 2011, four days after the earthquake [5,6]. The director-general of the Nuclear Emergency Response Headquarters ordered the evacuation of individuals around the FNPP. On March 16, 2011, the Japanese and prefectural government began to monitor select foodstuffs (e.g., milk, vegetables, grains, meat, fish). Foods containing radioactive material that exceeded the provisional regulation values (500 Bq/kg or 500 Bq/L) were prohibited from distribution on March 22, 2011 and were prohibited from consumption on March 23, 2011. These actions were taken to minimize the external and internal radiation exposure of the general population in Fukushima [1–3].

Fukushima Medical University Hospital (FMUH), located 60 km from the FNPP, was designated as a secondary radiation emergency medical care facility in Japan at the time of the accident. Although many medical staff including nurses worked intensively at FMUH during the radiation emergency, some nurses might have the intention to leave the job (ITL). However, there have been no studies on the percentage of nurses with ITL after the accident and the factors associated with nurses’ ITL.

Recently, Ohno et al. conducted a survey to establish an efficient training program on radiation safety for nurses. They found that nurses did not have enough knowledge of radiological treatment and did not know the impact of radiation on pregnant women [7]. They concluded that education programs in undergraduate school and at the workplace should be coordinated efficiently to ensure that both nurses and patients are informed about the meaning of radiation safety.

It is important to identify the factors associated with nurses’ ITL during radiation emergencies in order to prepare education programs for nurses working at radiation emergency medical care facilities and related public health centers. In this study, we conducted a survey among nurses who were working at FMUH at the time of the accident to clarify the factors associated with their ITL during the radiation emergency.

## Materials and Methods

### Study Participants

The study was conducted in June 2013 at FMUH, a tertiary medical facility of Fukushima Prefecture that was designated as a secondary radiation emergency medical care facility at the time of the FNPP accident. We initially distributed questionnaires to 537 nurses who were working at FMUH during the accident and were still working at the time of the study. We obtained responses from 479 nurses (89.2%). After excluding 68 nurses with insufficient responses and 66 nurses who answered that they had ITL before the accident, only 345 nurses (17 men and 328 women) were included in the analysis. The study was approved by the ethics committees of Nagasaki University Graduate School of Biomedical Sciences and FMUH.

### Questionnaire

The questionnaire for this study was developed based on our previous study [8], the Mental Health and Lifestyle Survey within the framework of the Fukushima Health Survey [9], and a Q&A that we published for residents of Fukushima Prefecture after the accident [10].

In the questionnaire, we asked about the nurses’ ITL within two months after the accident, within two months to one year after the accident, within one to two years after the accident, and at the time of the study. We also asked about the nurses’ demographic factors including sex, age at the time of the accident, tenure as a nurse, tenure as a nurse at FMUH, employment rank, marital status, pregnant status, experience working in the radiological examination and/or therapy section before the accident, and experience in nursing patients with radiation therapy.

We asked the questions about the nurses’ living situation, possible radiation health effects on the respondents, the children, and the residents of Fukushima, working status and their knowledge of radiation health effects. Refer to **Table 1, 2 and 3** for the list of questions.

**Table 1:** Nurses’ living situation by ITL after the FNPP accident.

Questions	ITL (+) (n = 154)	ITL (-) (n = 191)	p
Did you live apart from your family after the accident?	48 (31.2)	41 (21.5)	0.027
Did you feel anxiety about life in Fukushima City after the accident?	152 (98.7)	166 (86.9)	<0.001
Did you experience difficulties answering about radiation?	107 (69.5)	136 (71.2)	0.408
Did you measure the ambient dose rate around your house after the accident?	117 (76.0)	125 (65.4)	0.022
Did you think that the ambient dose rate around your house was relatively high after the accident?	143 (92.9)	160 (83.8)	0.007
Have you thought about evacuating Fukushima since the accident?	102 (66.2)	65 (34.0)	<0.001
Have you hesitated to buy vegetables produced in Fukushima since the accident?	139 (90.3)	147 (77.0)	0.001
Have you bought mineral water for drinking since the accident?	122 (79.2)	118 (61.8)	<0.001
Have you ordered your children not to play outside since the accident?	65 (92.9)	59 (83.8)	0.063
Do you currently feel anxiety about radiation exposure?	121 (78.6)	126 (66.0)	0.007

*Note*. Number who answered “yes” (%).

**Table 2:** Nurses’ working environment by ITL after the FNPP accident.

Questions	ITL (+) (n = 154)	ITL (-) (n = 191)	p
Did you feel that your workload increased after the accident?	78 (50.6)	70 (36.6)	0.006
Did you feel anxiety about your relationships with colleagues in the hospital after the accident?	34 (22.1)	14 (7.3)	<0.001
Did you feel anxiety about your relationships with patients in the hospital after the accident?	23 (14.9)	15 (7.9)	0.028

*Note*. Number who answered “yes” (%).

**Table 3:** Nurses’ knowledge about radiation health effects before the accident by ITL after the FNPP accident.

Questions	ITL (+) (n = 154)	ITL (-) (n = 191)	p
Have you attended the lecture on radiation health effects?	42 (27.3)	60 (31.4)	0.236
Did you know the differences between radiation, radioactivity, and radioactive substances?	47 (30.5)	70 (36.6)	0.140
Did you know about half decay of radioactive substances?	77 (50.0)	112 (58.6)	0.068
Did you know about natural background radiation?	110 (71.4)	145 (75.9)	0.206
Did you know about the annual dose limit for the general public?	20 (13.0)	22 (11.5)	0.400
Did you know about the annual dose limit for occupational exposure?	28 (18.2)	33 (17.3)	0.468
Did you know about the three principles of radiation protection?	85 (55.2)	104 (54.5)	0.488
Did you know about external radiation exposure?	71 (46.1)	96 (50.3)	0.255
Did you know about internal radiation exposure?	58 (37.7)	80 (41.9)	0.247
Did you know about deterministic effects?	16 (10.4)	27 (14.1)	0.189
Did you know about stochastic effects?	14 (9.1)	28 (14.7)	0.079

*Note*. Number who answered “yes” (%).

### Statistical Analysis

We defined ITL (+) as “nurses who intended to leave the job at any period after the accident” and ITL (-) as “nurses who did not intend to leave the job at any period after the accident.” We identified the factors associated with ITL after the accident using the Mann-Whitney U test and chi-square test. We used logistic regression analysis and calculated odds ratios (OR) to identify the factors independently associated with ITL after the accident. P values less than 0.05 were considered significant.

## Results

Among the study participants, 154 (44.6%) had ITL after the accident (ITL [+]), and 191 (55.4%) did not have ITL at any time after the accident (ITL [–]). Among the 154 ITL (+), 89 (57.8%) had ITL within two months after the accident, 72 (46.8%) had ITL within two months to one year after the accident, 57 (37%) had ITL within one to two years after the accident, and 63 (41%) had ITL at the time of the study.

**Table 4** shows that when compared with ITL (-), ITL (+) were significantly younger (32.2±6.9 vs. 35.7±8.6 years old, p<0.001) and had significantly shorter tenure as a nurse (9.9±6.8 vs. 13.5±8.6 years, p<0.001) and as a nurse at FMUH (8.0±6.3 vs. 11.2±8.2 years, p<0.001). In addition, the percentage of general nurses was significantly higher among ITL (+) than ITL (-) (96.1% vs. 88.0%, p = 0.005). The percentage of nurses who experienced nursing the patients with radiation therapy was significantly lower among ITL (+) than ITL (-) (58.4% vs. 73.3%, p = 0.003).

**Table 4:** Nurses’ demographic factors by ITL after the FNPP accident.

	ITL (+) (n = 154)	ITL (-) (n = 191)	p
Age, M±SD (years)	32.2±6.9	35.7±8.6	<0.001
Tenure as a nurse, M±SD (years)	9.9±6.8	13.5±8.6	<0.001
Tenure as a nurse at FMUH, M±SD (years)	8.0±6.3	11.2±8.2	<0.001
Female, n (%)	142 (92.2)	186 (97.4)	0.025
Rank (general nurse), n (%)	148 (96.1)	168 (88.0)	0.005
Experience in nursing the patients with radiation therapy, n (%)	90 (58.4)	140 (73.3)	0.003
Pregnant, n (%)	19 (13.4)	8 (4.3)	0.003
Married, n (%)	76 (49.4)	186 (97.4)	0.025

A significantly higher percentage of ITL (+) nurses than ITL (-) nurses were living with preschoolers (26.6% vs. 14.1%, p = 0.003), with preschoolers and elementary school students (39.6% vs. 29.3%, p = 0.03), and with preschoolers, elementary school students, and junior high school students (42.2% vs. 33.0%, p = 0.05). The following percentages were also significantly higher among ITL (+) than ITL (-): those who lived apart from their family after the accident (31.2% vs. 21.5%, p = 0.027), those who felt anxiety about life in Fukushima City after the accident (98.7% vs. 86.9%, p<0.001), those who measured the ambient dose rate around their house after the accident (76.0% vs. 65.4%, p = 0.022), those who thought the ambient dose rate around their house was relatively high after the accident (92.9% vs. 83.8%, p = 0.007), those who considered evacuating Fukushima after the accident (66.2% vs. 34.0%, p<0.001), those who hesitated to buy vegetables produced in Fukushima after the accident (90.3% vs. 77.0%, p = 0.001), and those who felt anxiety about radiation exposure at the time of the study (78.6% vs. 66.0%, p = 0.007) (**Table 1**).

Regarding the working environment after the accident, ITL (+) had significantly higher percentages than ITL (-) in terms of those who felt that their workload increased after the accident (50.6% vs. 36.6%, p = 0.006), those who felt anxiety about their relationships with colleagues in the hospital after the accident (22.1% vs. 7.3%, p<0.001), and those who felt anxiety about their relationships with patients in the hospital after the accident (14.9% vs. 7.9%, p = 0.028) (**Table 2**). The percentage who had knowledge on radiation health effects was relatively low for both groups, with no significant differences between the groups (**Table 3**).

Logistic regression analysis revealed that living with preschoolers (OR = 1.87, 95%CI: 1.02–3.44, p = 0.042), anxiety about life in Fukushima City after the accident (OR = 5.55, 95%CI: 1.18–26.13, p = 0.030), consideration of evacuation from Fukushima after the accident (OR = 2.42, 95%CI: 1.45–4.06, p = 0.001), consideration of the possible radiation health effects in children (OR = 1.90, 95%CI: 1.02–3.54, p = 0.042), and anxiety about relationships with colleagues in the hospital after the accident (OR = 3.23, 95%CI: 1.57–6.64, p = 0.001) were independently associated with ITL after the accident (**Table 5**).

**Table 5:** Logistic regression analysis for ITL.

Variables	Unit	OR	95% CI	p
Experience in nursing the patients with radiation therapy	Y/N	0.69	0.41–1.15	0.149
Living with preschoolers	Y/N	1.87	1.02–3.44	0.042
Anxiety about life in Fukushima City after the accident	Y/N	5.55	1.18–26.13	0.030
Relatively high ambient dose rate around the house	Y/N	0.82	0.33–2.02	0.668
Consideration of evacuation from Fukushima	Y/N	2.42	1.45–4.06	0.001
Consideration of the possible radiation health effects in children	Y/N	1.90	1.02–3.54	0.042
Increased workload after the accident	Y/N	1.59	0.98–2.57	0.061
Anxiety about relationships with colleagues in the hospital after the accident	Y/N	3.23	1.57–6.64	0.001

*Note*. OR = odds ratio, CI = confidence interval.

## Discussion

We found that several demographic factors including age and tenure as a nurse were associated with nurses’ ITL after the FNPP accident. Logistic regression analysis showed that tenure as a nurse was independently associated with ITL. Several studies have indicated that demographic factors such as being male, being single, and working the night shift are highly associated with ITL [11–17] and that nurses’ ITL decrease as education level increases [18]. We found that younger age and shorter tenure were significantly associated with ITL. Several studies have shown that nurses who had worked for less than five years at their place of employment were less likely to stay than those who had worked there longer [16, 17, 19]. However, Liou and Cheng reported that nurses over 35 years old and with more than 10 years of experience at their current hospital had greater ITL than other groups, whereas nurses under 25 years old and with less than one year of experience at their current hospital had less ITL [15]. Such inconsistent results may be due to the influence of other demographic factors. Thus, further studies are needed to clarify the effects of demographic factors on ITL.

We also found that factors related to the working environment, such as increased workload and anxiety about one’s relationships with colleagues and with patients, were associated with nurses’ ITL after the FNPP accident. Logistic regression analysis showed that the nurses’ anxiety about their relationships with colleagues in the hospital after the accident was independently associated with ITL. AbuAlRub et al. reported that nurses who perceived themselves as having more social support from supervisors and co-workers reported a higher level of intent to stay in both public and private hospitals [20]. Studies have also shown that good communication with peers is positively associated with the intention to stay in a job [11, 13]. Our results suggest that even during radiation emergencies, factors related to the working environment, including good relationships with colleagues, are important factors in nurses’ ITL. Nevertheless, the numbers of nurses who felt anxiety about their relationships with patients in the hospital after the accident were relatively small in both groups. Further studies are needed to clarify the contribution of the working environment to ITL during radiation emergencies.

In the present study, many factors related to the living situation were associated with ITL. These factors include a relatively high ambient dose rate, consideration of evacuation, and anxiety regarding external and internal radiation exposure, especially among children. Logistic regression analysis showed that anxiety about life in Fukushima after the accident and consideration of evacuation from Fukushima after the accident were independently associated with ITL. Note, however, that the percentage of nurses who had knowledge on radiation health effects was relatively low in both groups, with no significant differences between the groups. For example, only 12.5% of nurses knew about “deterministic effects,” and only 12.2% knew about “stochastic effects.” These findings suggest that anxiety related to the living situation, particularly regarding radiation exposure, is largely due to the nurses’ insufficient knowledge on radiation health effects.

The Fukushima Health Survey estimated the external radiation dose based on descriptions of self-reported behavior following the accident [9]. The survey covered 20.5% of two million residents of Fukushima Prefecture; among these residents, the external effective dose between March 12 and July 11, 2011 was estimated at less than 1 mSv in 62.0% of individuals, less than 2 mSv in 94.0%, less than 3 mSv in 99.4%, less than 4 mSv in 99.7%, and less than 5 mSv in 99.8%. Eleven individuals had doses greater than 15 mSv [21]. In addition, thyroid dose monitoring was conducted from March 26 to March 30, 2011, using a NaI (Tl) scintillation survey meter. A total of 1,080 children under the age of 15 were measured in Iwaki City, Kawamata Town, and Iitate Village in Fukushima Prefecture. The results showed that 55% had only background radiation levels or lower, and 99% had levels below 0.04 μSv/h, which is equal to 20 mSv of a thyroid equivalent dose [22]. These findings suggest that external and internal doses from the FNPP accident were relatively limited in the general population.

Nevertheless, many nurses considered evacuating Fukushima after the accident; this was closely associated with their ITL after the accident. Radiation health risk communication is needed in Fukushima to avoid misunderstandings about radiation health effects. Nurses, especially public health nurses, should play a central role in this communication.

This study has several limitations. It was not a multi-institutional study, and we were unable to include nurses who had already left after the accident but prior to the study. These factors might have introduced selection bias in the choice of study participants. The relatively wide confidence intervals in the multiple regression analysis might also indicate considerable uncertainty in the results, probably due to insufficient numbers of study participants. Further studies are needed to clarify the factors associated with nurses’ ITL during radiation emergencies.

In conclusion, it is important for nurses to obtain the correct knowledge about radiation health effects. Thus, an education program for nurses on radiation health effects must be established in countries that have nuclear power plants and other nuclear facilities.
